# Targeted Oligodendrocyte Apoptosis in Optic Nerve Leads to Persistent Demyelination

**DOI:** 10.1007/s11064-019-02754-z

**Published:** 2019-03-08

**Authors:** Ahdeah Pajoohesh-Ganji, Robert H. Miller

**Affiliations:** Department of Anatomy and Cell Biology, The George Washington University School of Medicine and Health Sciences, Washington, DC 20037 USA

**Keywords:** Optic nerve, Oligodendrocytes, Demyelination, Apoptosis

## Abstract

The optic nerve represents one of the simplest regions of the CNS and has been useful in developing an understanding of glial development and myelination. While the visual system is frequently affected in demyelinating conditions, utilizing the optic nerve to model demyelination/remyelination studies has been difficult due to its accessibility, relatively small size, and dense nature that makes direct injections challenging. Taking advantage of the lack of oligodendrocytes and myelination in the mouse retina, we have developed a model in which the induction of apoptosis in mature oligodendrocytes allows for the selective, non-invasive generation of demyelinating lesions in optic nerve. Delivery of an inducer of oligodendrocyte apoptosis by intravitreous injection minimizes trauma to the optic nerve and allows for the assessment of oligodendrocyte death in the absence of injury related factors. Here we show that following induction of apoptosis, oligodendrocytes are lost within 3 days. The loss of oligodendrocytes is associated with limited microglial and astrocyte response, is patchy along the nerve, and results in localized myelin loss. Unlike in other regions of the murine CNS, where local demyelination stimulates activation of local oligodendrocyte precursors and remyelination, optic nerve demyelination induced by oligodendrocyte apoptosis fails to recover and results in persistent areas of myelin loss. Over time these chronic lesions change cellular composition and ultimately become devoid of GFAP+ astrocytes and OPCs. Why the optic nerve lesions fail to repair may reflect the lack of early immune responsiveness and provide a novel model of chronic demyelination.

## Introduction

Demyelinating diseases such as multiple sclerosis (MS) are characterized by lesions in CNS white matter where myelin and oligodendrocytes have been lost, and in most active lesions there is an infiltration of cells of the peripheral immune system [1, 2]. The majority of current therapies for MS are directed towards regulating the influx and function of the immune cells and while such treatments have benefit early in the disease, they ultimately fail to control chronic disease in most cases [3]. Such findings suggest that while inflammation is clearly a very important component of the pathogenesis of demyelinating diseases, other factors contribute to disease induction and progression. To provide insights into the mechanisms mediating CNS demyelination and to develop potential therapeutic approaches for MS and related diseases, a number of animal models have been established. In general, these fall into two distinct categories: those that utilize the induction of immune mediated damage to CNS white matter [4] and those that utilize a gliotoxin to drive local demyelination [5]. For example the most commonly used immune-mediated model of MS is experimental allergic encephalomyelitis (EAE) where injection of specific peptides of myelin proteins such as MBP, PLP or MOG into a susceptible host genotype combined with stimulation of the immune system results in wide spread infiltration of immune cells and myelin loss [4]. This model has been particularly useful in identifying therapies that affect the immunological component of MS but less effective in identifying therapies that influence the neuroprotection and remyelination components of MS. Gliotoxin models such as cuprizone toxicity, induced by feeding chow containing copper chelator cuprizone [5, 6] and local injection of lysolethicin [7, 8] or ethidium bromide [9] allow for analysis of modulators of the process of myelin repair [10]. Although these models have provided critical insights into the demyelination/remyelination processes, they have some limitations. For example, generation of the EAE models requires a strong immune stimulus that is likely missing in natural disease. Likewise, the mechanism of induction of the gliotoxin models is clearly not physiological and may influence multiple cellular and molecular targets that affect repair.

One issue not easily resolved by the immune and gliotoxin models is whether the onset of CNS demyelination is a reflection of immune cell infiltration that drives oligodendrocyte death and demyelination or whether oligodendrocyte death results in demyelination and immune cell infiltration. To begin to address this issue, we have utilized transgenic mice in which oligodendrocytes are specifically targeted to undergo apoptosis utilizing an inducible form of caspase 9 (termed iCP9) linked to a DsRed reporter targeted to mature oligodendrocytes by a fragment of the myelin basic protein promoter (p-MBP). Myelin basic protein is selectively expressed by mature oligodendrocytes in the CNS and is a major component of CNS myelin [11, 12]. Previously, we have shown that dimerization of iCP9, by a cell permeable small molecule cross-linker related to FK506 (Chemical Inducer of Dimerization CID), which penetrates the brain and the spinal cord, triggers the apoptotic pathway in MBP-iCP9 animals resulting in selective death of MBP+ oligodendrocytes while leaving non-MBP+ cells untouched [11]. For example, developmental studies have shown that delivery of CID to the spinal cord during the first postnatal week results in a transient demyelination period, which fully recovers within 2 weeks [13]. This model has a number of strengths. The mechanism of induction of cell death is physiological, since a subset of oligodendrocytes and their precursors are known to undergo apoptosis during development [14–17] and the targeting of cell death is specific to oligodendrocytes, avoiding the complication of damage to other cell types. Although apoptosis may not be the only mode of oligodendrocyte loss in MS, in this model oligodendrocyte loss clearly occurs prior to any engagement of the immune system allowing unambiguous characterization of downstream outcomes.

In the current study we use MBP-iCP9 transgenic mice to examine demyelination and remyelination in the optic nerve. The optic nerve was important for initially defining the pathways of glial cell development because it is one of the simplest regions of the CNS and lacks neurons [18–20]. The vast majority of axons in the optic nerve is derived from retinal ganglion cells and is relatively uniform in size. Early studies demonstrated separate lineages and origins of astrocytes and oligodendrocytes in the optic nerve [19]. While the majority of optic nerve astrocytes arise from cells of the optic stalk, the majority of oligodendrocytes are derived from progenitor cells that migrate into the nerve from the floor of the third ventricle [21, 22]. The visual system and optic nerve are frequently implicated in demyelinating diseases and optic neuritis is an initial symptom of MS observed in a quarter of diagnosed cases [23]. Recent clinical trials of remyelinating therapies have also focused on the visual system to assess repair [24]. However, it has been challenging to use the optic nerve in animal models of demyelination and repair. This is due in part to the difficulty of access to the nerve for surgical procedures as well as the challenge that attempts to directly deliver toxins or therapies to the nerve often result in axonal damage. To circumvent these concerns, we have developed an approach to deliver CID to the optic nerve indirectly through an intravitreous injection at the limbus. Using this approach, we show that small molecules successfully pass through the lamina cribrosa and permeate the length of the nerve. Following delivery of CID to MBP-iCP9 animals, oligodendrocyte death is seen within 2–3 days. Adjacent astrocytes and microglial cells are unaffected and have no apparent response to oligodendrocyte loss and the myelin sheaths are minimally affected. 1 week after CID delivery, demyelination is detectable along the nerve in discrete patches. Over the following week, microglia become activated and areas of demyelination continue to develop. At 3 and 4 weeks post-CID, demyelination persists, microglial responses diminish, and the expression of GFAP and potentially the number of astrocytes in the lesions decreases. These findings suggest that demyelination of the optic nerve induced by oligodendrocyte apoptosis results in lesions that fail to rapidly repair and may be used as a model for chronic demyelinating disease.

## Materials and Methods

### Animals and In Vivo Injections

All studies performed comply with the George Washington University Medical Center Institutional Animal Care and Use Committee guidelines. Both male and female MBP-iCP9 transgenic mice [11] on a C57Bl6 background were used. Due to the limited number of transgenic pups, we were unable to perform gender specific studies.

Two weeks old pups were injected intravitreously at the limbus under inhaler anesthetic (Isoflurane) for 3 consecutive days. A 23 3/8 gage needle at 45° angle was inserted at the limbal region to create an injection site and a sterile Q-tip was used to absorb the vitreous fluid flow before injection. 1 µL of 5 mM CID (clontech laboratories; #635069) was made with 1XPBS from 50 mM CID in 100% ethanol stock solution and was delivered to the right eye at a rate of 60 nl/s using a glass pipet. The pipet was left at the injection site for an additional minute to minimize CID leakage. The injection site was covered with ophthalmic antibiotic ointment to prevent CID leakage and infection. The left eye was used as an internal control. Under terminal sedation, mice were transcardially perfused with 1xPBS followed by fixative at 1 day or 1, 2, 3, or 4 weeks after the first injection and optic nerves attached to the retina were dissected. Tissues were fixed overnight with either 4% paraformaldehyde for immunofluorescence staining or 4% paraformaldehyde-2% glutaraldehyde-0.1 M sodium cacodylate for electron microscopy. A minimum of three animals were used at each time point.

### Tissue Processing

For *Immunofluorescence staining*, longitudinal or cross sections were cut using stereological techniques to assure random sampling from tissues that were cryoprotected using 10, 20, and 30% sucrose gradient in 1 × PBS solutions. Cross sections 1–18 were collected on each slide starting at the retina and ending at the optic chiasm with a 300 µm interval between individual sections. Sections were rehydrated with 1 × PBS for 5 min and blocked for 1 h at room temperature. They were incubated with the primary antibodies overnight followed by appropriate secondary antibody incubation for an hour. Sections were then counterstained with Dapi, mounted, and coverslipped. Some sections were stained with Toluidine Blue staining to visualize myelin. For *semi-thin tissues*, tissues were osmicated (1% OsO_4_) for at least 4 h followed by 1% uranyl acetate overnight. After gradated dehydration with ethanol and propylene oxide, the tissues were placed in Epon812 and cut at 1 µm or 70 nm with a microtome. The 1 µm sections were stained with a solution of 1% toluidine blue/1% sodium borate for about 2 min.

### Microscopy

Confocal microscopy was performed at the Center for Microscopy and Image Analysis (CMIA) at the George Washington University Medical Center. A confocal laser-scanning microscope (Zeiss 710) equipped with a krypton-argon laser was used to image the localization of Alexa Fluor 488 (490 excitation max; 525 emission max) or Alexa Fluor 594 (590 nm excitation max; 617 emission max). Optical sections (z = 0.5 µm or 1 µm) were acquired sequentially with a × 63, or × 100 objective lens. Images presented were generated using Volocity software (Version 6.3, Perkin Elmer). For broad filed images taken from the entire optic nerve, tile images were acquired with a multi-immersion corrected objective lens (i.e., × 25/0.8, 560 µm working distance).

### Antibodies

Sections were stained with the following antibodies: MBP (1:300—Abcam; #7349), DsRed (1:400—Takara; #632496), Iba1 (1:500—WACO; #019-19741), GFAP (Biolegend PRB-571C), S100 (1:500—DAKO; #Z0311), Ki67 (1:200—Abcam; ab16667), PDGFαR (1:100—BD Biosciences; #558774), and CC1 (1:200—Millipore; #OP80). Appropriate secondary Alexa 488 or 594 (1:500) antibodies were used. Sections were stained with DAPI (1:1000—Thermo fisher; #46190) before mounting (Fluoromount G: Electron Microscopy Sciences; #17984-25) to visualize the nuclei.

### Statistical Analyses

All statistical tests were performed using the GraphPad Prism Program, Version 6 (GraphPad Software, Inc. San Diego, CA). A p value < 0.05 was considered statistically significant and is shown with an asterisk. NIH Image J was used to analyze the total and demyelinated areas of the optic nerves. Photoshop was used to count cells.

## Results

### Intravitreous Delivery of Drugs Allows for Non-invasive Access to the Optic Nerve

**Fig. 1:**
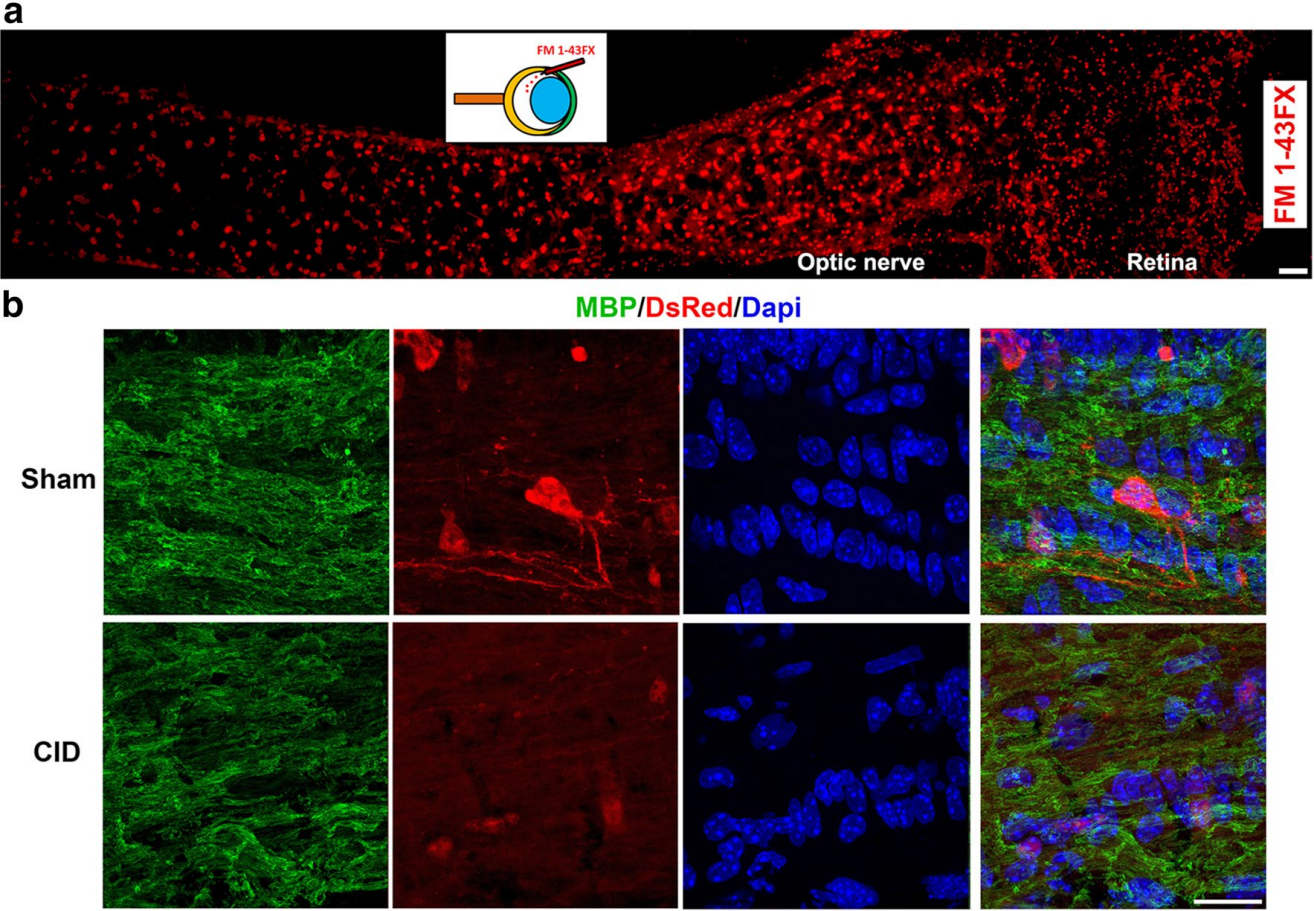
CID intravitreous injection leads to oligodendrocyte apoptosis in the optic nerve. **a** Intravitreous delivery of small molecules results in broad dispersal along the optic nerve. Injection of the dye (FM 1-43FX) demonstrates rapid penetration of the dye from the retina into the optic nerve within 1 h. The inset depicts the angle of injection into the vitreous at the limbus (Bar = 50 µm). **b** Intravitreous delivery of CID into MBP-iCP9 transgenic animals results in loss of DsRed oligodendrocytes. Animals were injected with CID in the right eye and the left eye was the sham control. Mice were sacrificed 2 days after the first injection and longitudinal sections are stained with MBP (green) and DsRed (red) to visualize myelin and oligodendrocytes, respectively; Dapi (blue) stains the nuclei. In control (sham) nerves extensive DsRed+ oligodendrocyte cell bodies and processes where detectable. By contrast following CID injection only fragments of DsRed+ cell bodies and processes remain. Bar = 10 µm

The major challenges in utilizing the optic nerve for studies of demyelination/remyelination are the difficulties of surgical access and the risk of axonal damage following direct injections. To circumvent these issues, we have refined the approach of drug delivery to optic nerve through intravitreous injection at the limbus (Fig. 1a). To assess the extent of dispersal of intravitreal delivered compounds along the optic nerve, FM 1-43FX dye was injected into the eye and its distribution along the nerve assessed after 24 h. Labelling was present in the retina, optic nerve head and along the length of the nerve to the chiasm. The degree of dye labeling was similar throughout the nerve and the nerve appeared undamaged indicating that injection in the vitreous provides an effective conduit for drug delivery to the intact optic nerve.

Using the intravitreous delivery approach, the capacity to selectively ablate mature oligodendrocytes in the optic nerve has been examined. Recent studies demonstrated the ability to selectively ablate oligodendrocytes in the CNS of a novel transgenic mouse line in which an inducible form of caspase 9 (iCP9) linked to a DsRed reporter is driven by a fragment of the myelin basic protein promoter (pMBP) [11]. These (MBP-iCP9) animals have normal numbers of oligodendrocyte lineage cells, approximately 70% of which express the DsRed reporter during the first 2 months of life. Dimerization of the iCP9 construct through delivery of CID stimulates the apoptotic pathway resulting in oligodendrocyte death [13]. Intravitreous injection of CID into MBP-iCP9 animals resulted in the rapid death of DsRed+ oligodendrocytes [12]. Forty-eight hours after CID injection, the number of DsRed oligodendrocytes in the optic nerve was reduced compared to sham nerves and many of the residual cells showed fragmented processes and shrunken cell bodies (Fig. 1b). Dapi labeling suggested that the overall cell density in treated nerves was also reduced. Myelin was present along the nerve in both sham and CID injected eyes, although the integrity of the myelin appeared somewhat less uniform in CID treated animals (Fig. 1b) suggesting that oligodendrocyte apoptosis results in rapid changes in myelin organization. While the number of DsRed+ cells was significantly reduced following CID treatment, analysis of GFAP expression indicated no substantial changes in the number or morphology of optic nerve astrocytes (data not shown). These data demonstrate the ability to selectively ablate mature oligodendrocytes in the optic nerve without inducing direct axonal trauma.

**Fig. 2:**
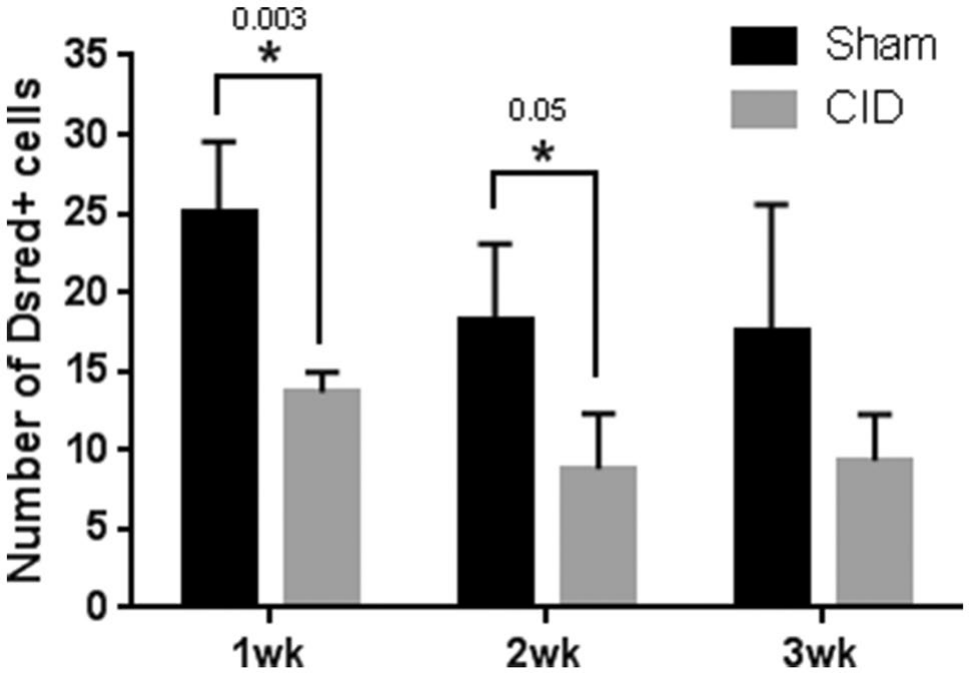
Persistent loss of DsRed+ cells following CID injection. Quantitation of the relative number of DsRed+ oligodendrocytes taken from cross sections of the optic nerve following CID injection. Significant reductions in the number of DsRed+ oligodendrocytes were observed at 1 (p value = 0.003) and 2 (p value = 0.05) weeks after CID injection compared to controls. The same trend is observed at 3 weeks after treatment, suggesting a lack of rapid recovery of the DsRed+ cells

In other regions of the CNS, demyelination and oligodendrocyte loss induced by LPC injection promotes the rapid response of OPCs to initiate repair [13]. To determine whether DsRed cells were replaced following CID induced ablation in MBP-iCP9 animals, the number of Dsred+ cells were compared at 1, 2, and 3 weeks after CID injection to sham eyes (Fig. 2). 1 week after CID induced oligodendrocyte ablation, the relative number of DsRed+ cells was significantly reduced compared to controls and this reduction persisted for up to 3 weeks (p value = 0.003 at 1 week and p value = 0.05 at 2 weeks) after CID injection, suggesting a lack of rapid recovery of DsRed cells after optic nerve ablation. Although not significant, the number of DsRed+ cells in control nerves decreased at 2 and 3 week interval likely due to reduced MBP promoter activity.

### Oligodendrocyte Ablation Results in Slow Onset Optic Nerve Demyelination

**Fig. 3:**
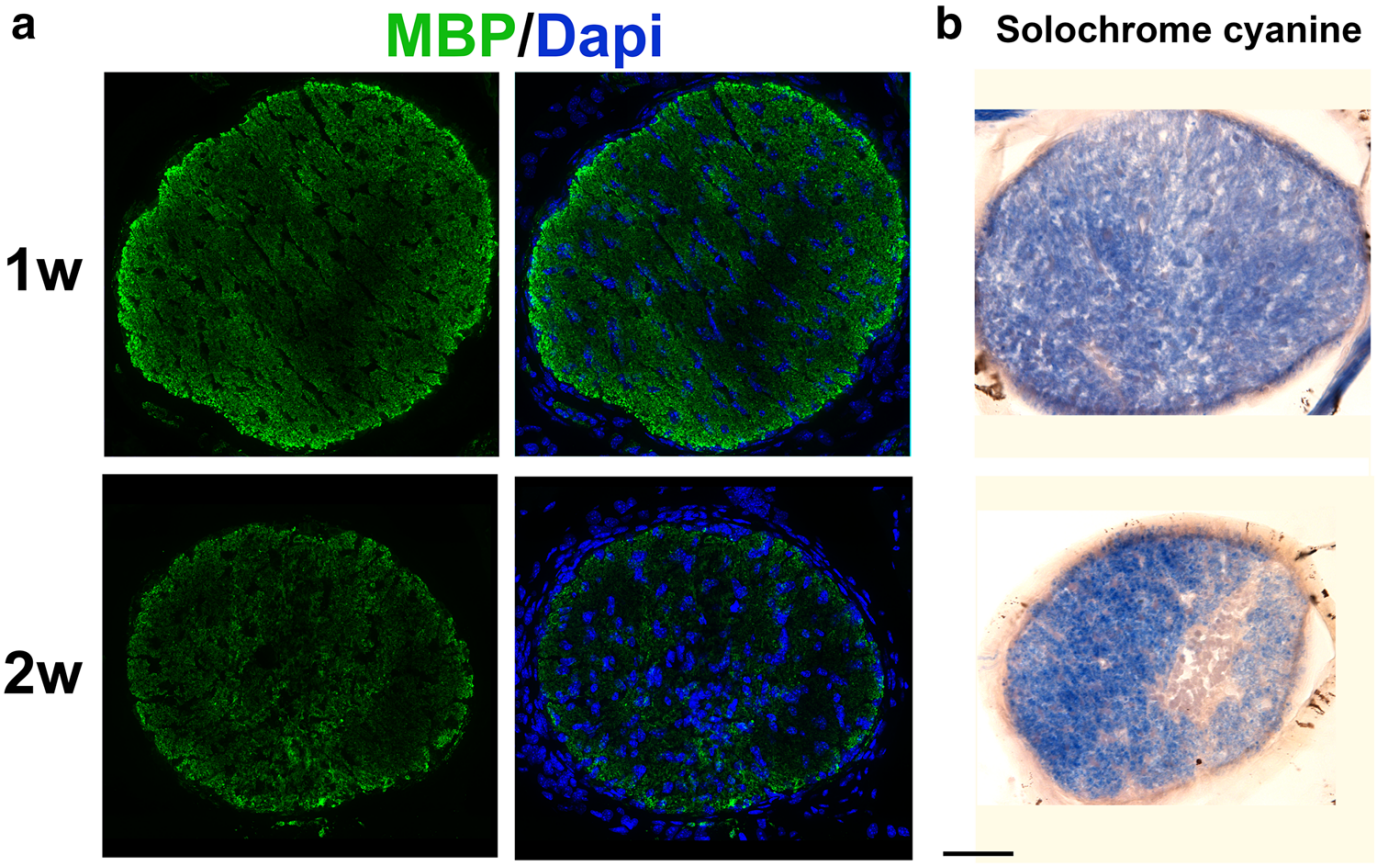
Demyelination of the optic nerve is detectable 1 week after CID treatment. **a** Cross sections of optic nerve were labeled with antibodies against myelin basic protein. Areas of demyelination were detectable as reduced MBP expression (green) at 1 and 2 weeks after CID injection. At 1 week post CID, loss of myelin was relatively diffuse in both **a** MBP immunohistochemistry and **b** solochorme cyanine labeling. At 2 weeks post CID, the nerves appeared thinner, the areas of demyelination were more pronounced and an enhanced cellularity was present in regions of myelin loss. Bar = 50 µm

**Fig. 4:**
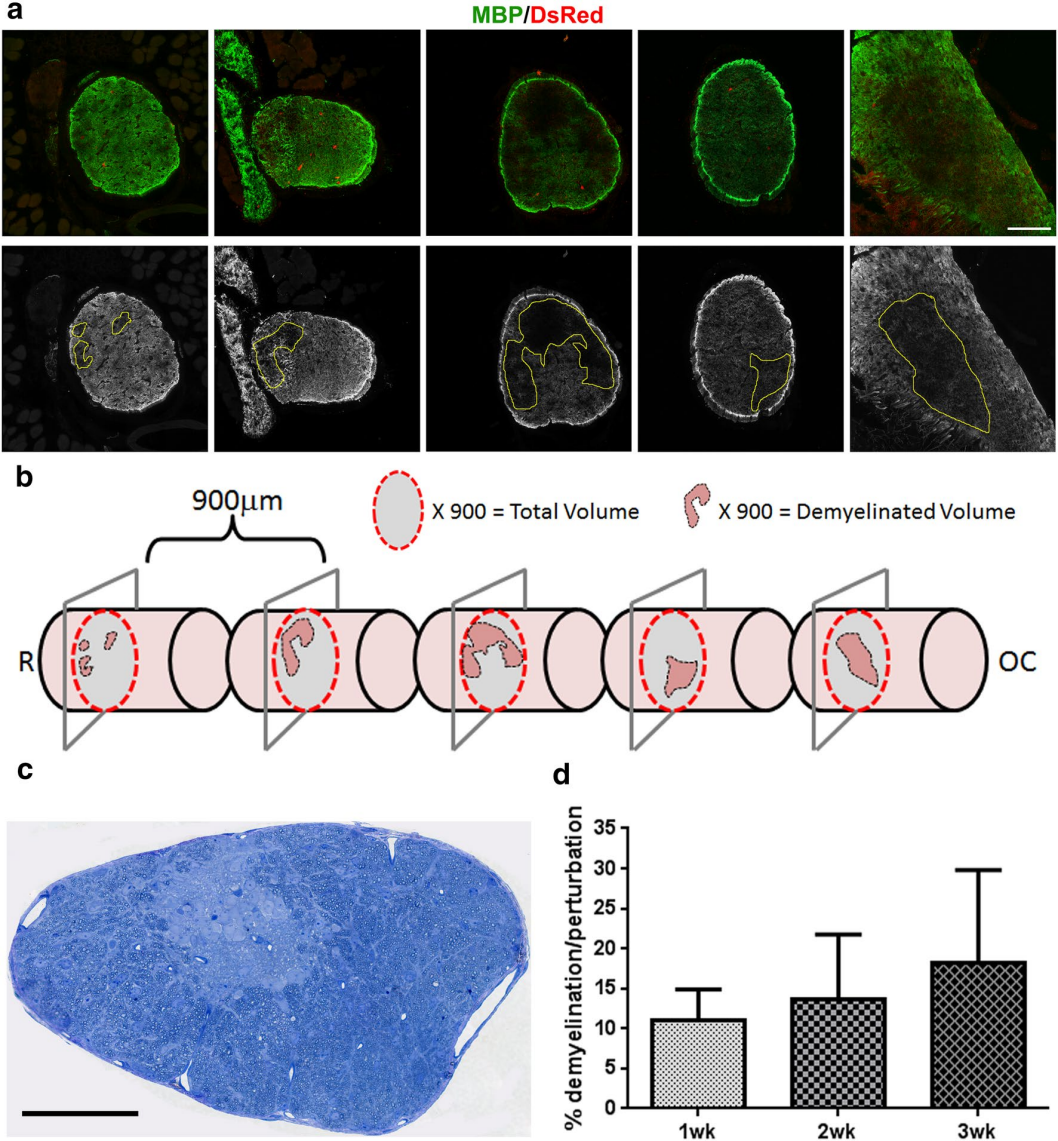
Demyelination induced by oligodendrocyte apoptosis does not repair rapidly and lesion volumes expands over time. MBP-iCP9 transgenic mice were injected with CID and sacrificed at 1, 2, or 3 weeks after the first injection. **a** Cross sections from CID-treated nerves 3 weeks after CID injection were cut using stereological methods and stained with MBP (green) and DsRed (red) to quantify change in demyelination. The MBP images were then converted to grayscale and the total nerve and demyelinated areas were measured using image J software and volumes were calculated. **b** 3D cartoon based on the sections depicts the patchy nature of the demyelination. **c** Toluidine blue staining of semi-thin section demonstrates distinct regions of myelin loss in CID treated nerves. **d** Percent demyelination was calculated at different time points indicating that the lesion does not repair up to 3 weeks after oligodendrocyte ablation. Bar = 100 µm in **a** and **c**

To determine whether the loss of DsRed+ oligodendrocytes results in optic nerve demyelination, the expression of MBP was compared in sham and CID injected MBP-iCP9 optic nerves at 1, 2 and 3 weeks post injection. In sham nerves, intense MBP expression was uniformly present throughout the nerve and along its entire length (data not shown). In contrast to 2 days post CID nerves that had limited myelin perturbation, 1 week post-CID regions of the nerve showed reduced levels of MBP expression suggesting late onset of demyelination. The reduction in MBP labeling and perturbation of myelin organization was increasingly apparent at 2 and 3 weeks post CID (Figs. 3a, 4a) with 80% of nerves (4/5) demonstrating perturbations in MBP expression. At all ages the demyelination was not uniform in the nerve, but rather appeared as patches. Increased cellularity was observed in some regions of reduced MBP expression, suggesting a response by adjacent neural cells. Analysis of 20 µm sections stained with solochrome cyanine confirmed that areas with reduced MBP expression represented regions of demyelination (Fig. 3b) while in sham animals, myelinated axons were densely packed and surrounded by fine glial processes in all regions of the nerve. Semi-thin sections from 3 week post-CID treated nerves stained with toluidine blue showed variations of myelination throughout the nerve: some regions of the nerve appeared relatively normal, in other regions myelinated axons were more loosely packed and surrounded by more pronounced glial processes, and some regions of the nerve were devoid of myelinated axons. The regions of demyelination contained axonal profiles that were similar in diameter to adjacent myelinated axons. In other regions of the CNS, such as spinal cord, remyelinating axons are characterized by abnormally thin myelin sheaths. In the regions of optic nerve demyelination, no thinly myelinated axons were detectable and there was a relatively sharp boundary between the demyelinated and myelinated tissue (Fig. 4c).

### Oligodendrocyte Ablation Results in Chronic Demyelination that Does Not Repair

**Fig. 5:**
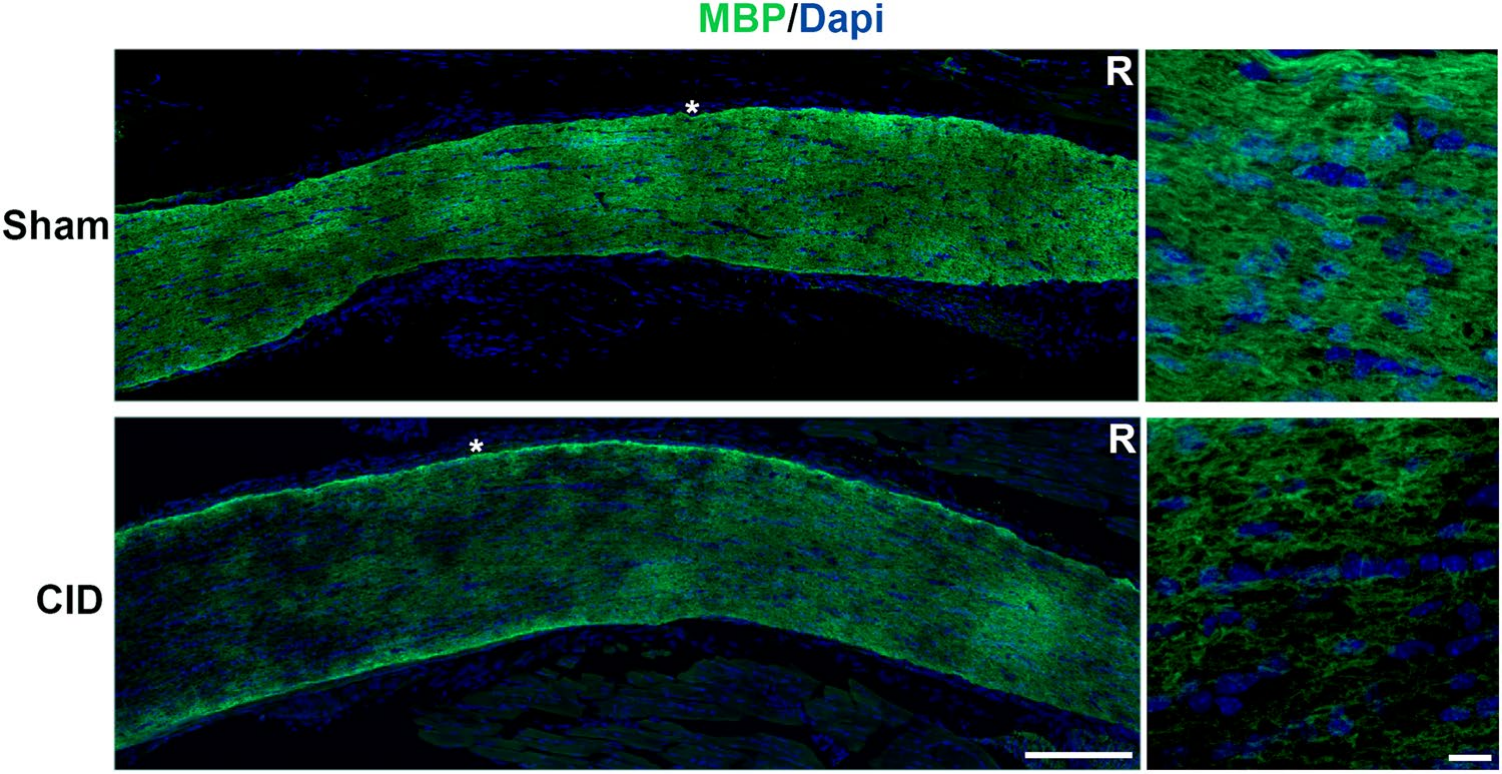
Demyelination persists 4 weeks after CID injection. Longitudinal sections were cut and stained with anti-MBP (green) and Dapi (blue) 4 weeks after CID injection. In the left panel, loss of myelin is apparent as dark patches in the CID injected optic nerve, which increases with distance from the retina (R). Regions marked with the asterisks are shown at higher magnification in the right panels. Bar = 10 µm

To accurately assess the extent of demyelination post-CID, stereological techniques were used to cut sections and quantify the demyelination present in the optic nerves at 1, 2, and 3 weeks post-CID. Sections were cut at 900 µm intervals and labeled with antibodies to MBP and DsRed. Images stained with MBP were converted to grayscale images (Fig. 4a) and optic nerve volumes were calculated by multiplying the area of MBP staining by the appropriate intervals. Demyelinated and total volumes for each nerve were calculated by adding the volumes together. To obtain the proportion of the nerve with demyelination, the demyelinated region was divided by the total volume of optic nerve. A 3D reconstruction of an optic nerve 3 weeks post-CID reveals the patchy nature of myelin loss along the nerve (Fig. 4b). Quantitation of the extent of demyelination indicated that rather than undergoing repair during a 21 day post lesion interval, the lesion area continued to expand from around 10% at 7 days to > 25% at 21 days post CID (Fig. 4d; Table 1). The extent of myelin loss was not uniform along the nerve. Comparative analysis demonstrated that the regions towards the chiasm had more pronounced myelin loss than those closer to the retina. Extending the analysis to 4 weeks post-CID failed to provide an indication of myelin repair. Myelin was present throughout the length of the nerve in sham animals. By contrast, post-CID treated nerve had a patchy expression of MBP that appeared worse towards the proximal end of the nerve close to the optic chiasm (Fig. 5). Taken together these studies indicate that induction of oligodendrocyte apoptosis in the optic nerve results in a slowly developing patchy demyelination that fails to repair even with extended post lesion intervals.

**Table 1:** The extent of demyelination was increased with time after CID injection

Weeks after first CID injection	Percent demyelination
1	15.3
1	10.1
1	7.9
2	16.0
2	8.4
2	6.1
2	24.0
3	4.4
3	32.2
3	17.2
3	11.1
3	12.1

MBP-iCP9 transgenic mice were injected with three consecutive injections of CID in the right eye; the left eye was used as control. Mice were sacrificed at 1, 2, and 3 weeks after the first injection. Percent demyelination was calculated using grayscale images utilizing image J as shown in Fig. 4. While the level of myelin loss was somewhat variable between nerves, the mean area of myelin loss was 11% at 1 week, 14% at 2 weeks and 16% at 3 weeks, demonstrating a failure of rapid repair

### Muted and Delayed Microglial and Astrocytic Response Combined with Limited OPC Proliferation Contribute to Lack of Remyelination

**Fig. 6:**
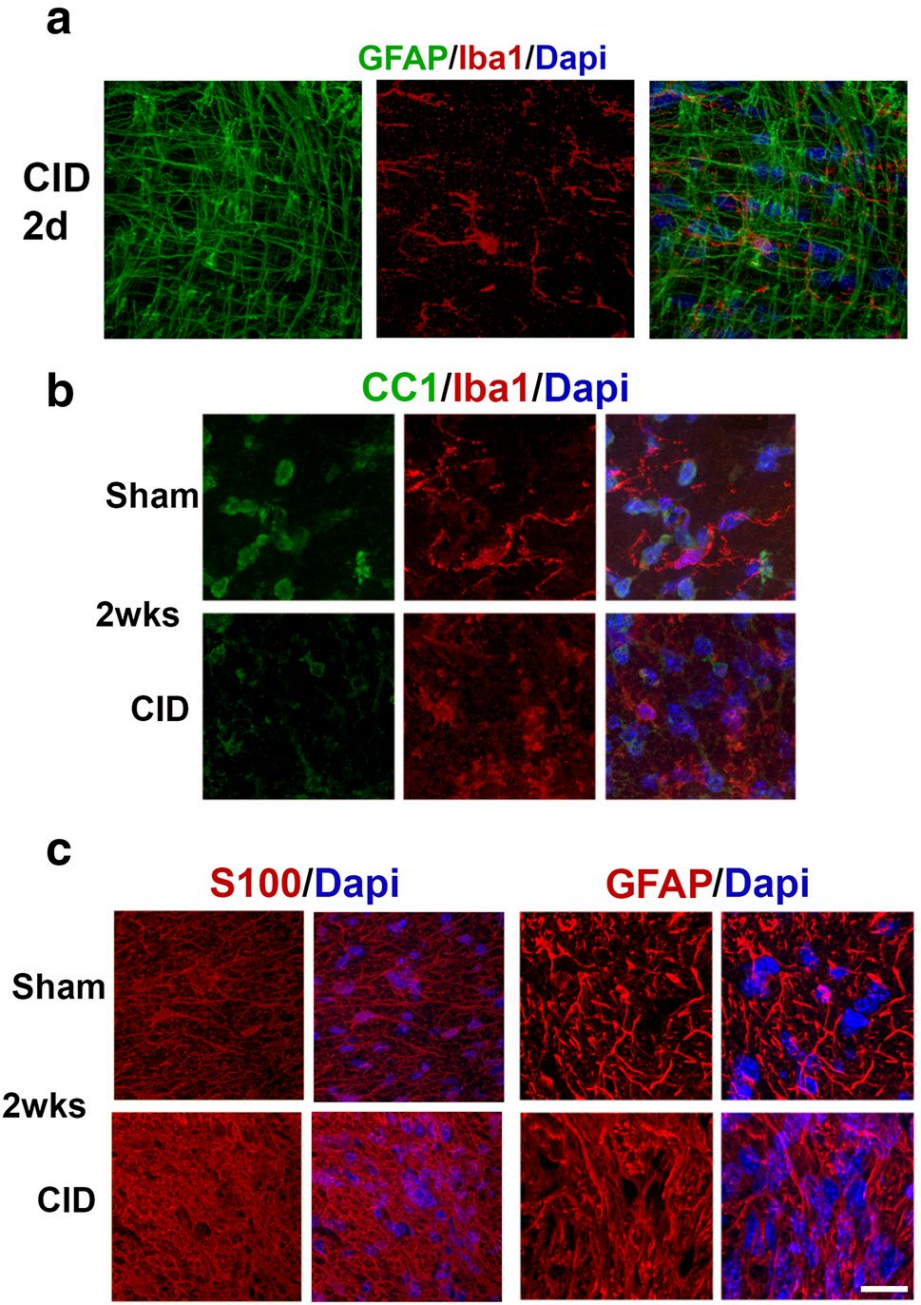
Oligodendrocytes ablation fails to stimulate an early robust immune response. **a** Sections labeled for microglia (Iba1, red) and Dapi (blue) 2 days after CID injection showed little microglial activation at this time point. **b** At 2 weeks after CID injections, there was a decrease in the number of mature oligodendrocytes and an increase in microglia compared to controls. Cross **s**ections of optic nerve were labeled for mature oligodendrocytes (CC1, green) and microglia (Iba1, red). **c** At 2 weeks after CID injection, there is an increase in S100 and GFAP+ astrocytes (GFAP, red) and nuclei (Dapi, blue) compared to controls. Bar = 10 µm

**Fig. 7:**
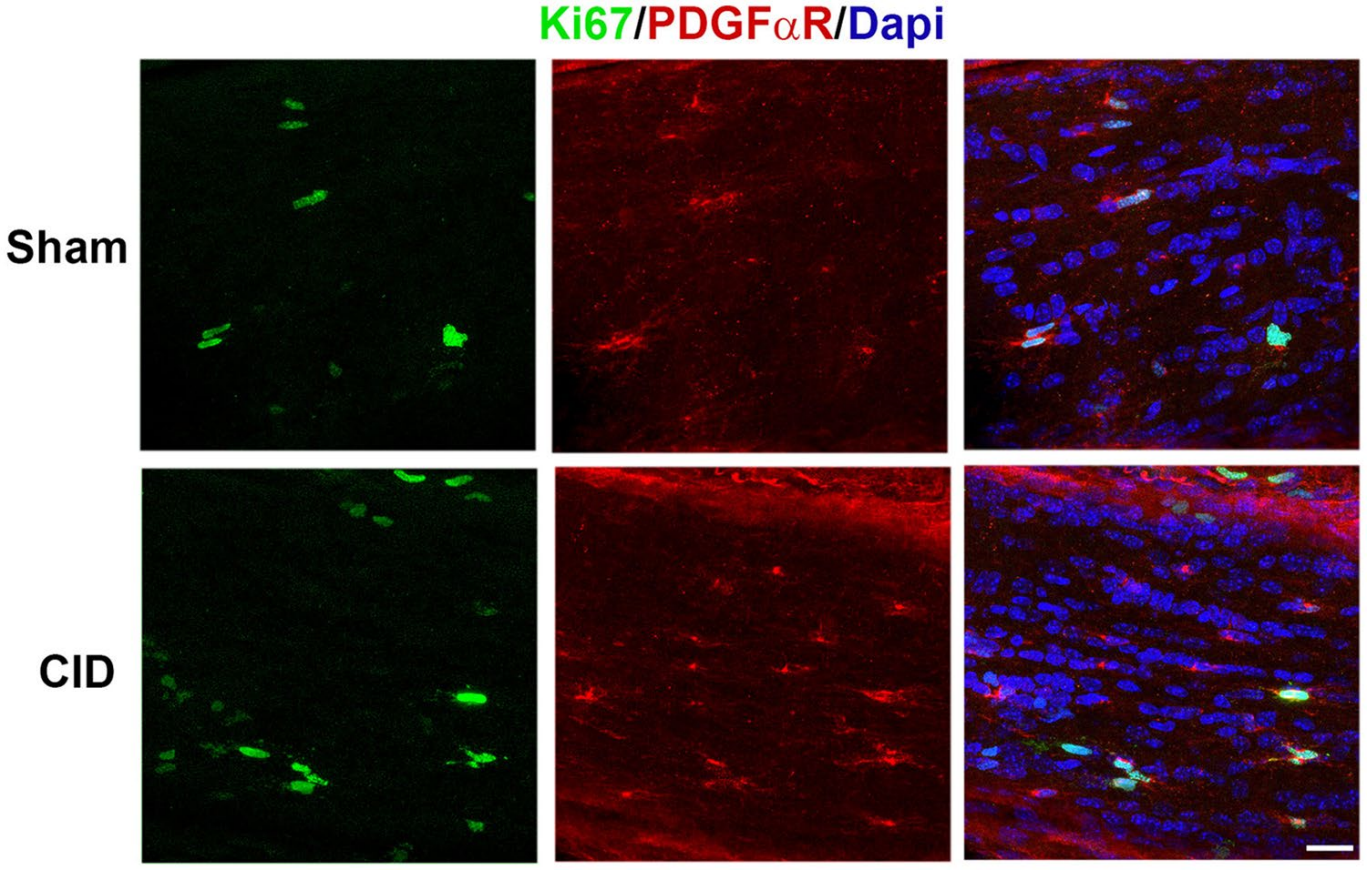
Oligodendrocyte progenitor cells have a muted response after CID treatment. Sections from 1 week post CID and sham nerves double labeled with antibodies to Ki67, green, and PDGFαR, red, show only a slight increase in the proliferation of OPCs following oligodendrocyte ablation. The total number of cells is also not substantially increased as shown by Dapi (blue). Bar = 20 µm

The observations that in the early stages of MS and in many animal models, active demyelination is associated with a robust innate inflammatory response that may contribute to the stimulation of remyelination led us to assess the innate inflammatory and astrocyte response in non-remyelinating lesions in the optic nerve. At 2 days post-CID in MBP-iCP9 optic nerves, the reduction in the number of DsRed+ cells was associated with only subtle changes in myelin organization and no obvious differences in astrocyte or microglial morphology suggesting that oligodendrocyte apoptosis fails to trigger rapid innate immune response (Fig. 6a). Similarly, at 1 week post-CID, although there was a significant loss of myelin in some regions of the optic nerve, there was limited response in astrocytes and microglia as assayed by GFAP and IBA1 expression (data not shown). Consistent with the loss of myelin, there was a significant reduction in the relative number of CC1+ oligodendrocytes in 2 weeks CID treated nerves (Fig. 6b, p value 0.006). However, unlike many other lesion models, the response of OPCs to the demyelination was very limited. The number of PDGFαR+ OPCs was slightly elevated in CID treated nerves at 1 week, which corresponded to a slight increase of Ki67 proliferating OPCs (Fig. 7) and was decreased at 2 and 3 weeks after CID treatment. Quantitation of lesion load indicates that the proportion of demyelination continues to increase over a 3 week period in the current model (Fig. 4d) and microglial and astrocyte responses peak at 2 weeks post-CID (Fig. 6b, c). Astrocyte reactivity decreases in the demyelinated areas and correlates with reduction in myelin levels and levels of MBP expression. By 3-weeks post-CID, microglial response and morphology are relatively normal even in areas of demyelination (data not shown). This reduction in GFAP expression was correlated with a reduction in the total cell number in the nerve as shown by Dapi labeling as well as a reduction in the overall size of the nerve. Taken together these data suggest that demyelination of the optic nerve, generated by local induction of oligodendrocyte apoptosis, fails to stimulate a significant innate immune response that in turn fails to stimulate a robust remyelination response.

## Discussion

The optic nerve has been used effectively to understand some of the principles of glial development due to its unique anatomy and ease of isolation [25]. Use of the optic nerve as an in vivo model for demyelination and remyelination studies has been more challenging due to difficulties with access and surgical approaches. Here we show that in a novel MBP-iCP9 transgenic mouse line, intravitreal delivery of an inducer of oligodendrocyte apoptosis results in slow optic nerve demyelination that fails to repair. While the loss of oligodendrocytes occurs within 2 days, the development of demyelination is relatively slow. Demyelination is apparent 1 week after CID delivery and persists for up to 4 weeks. The loss of oligodendrocytes is not associated with a substantial response in adjacent astrocytes or microglia, although there is some microglial and astroglial activation 2 weeks post-CID. The areas of demyelination are patchy along the length of the nerve and show no evidence of remyelination even with extended post-lesion intervals. Not only does localized oligodendrocyte apoptosis in the optic nerve fail to stimulate a response in adjacent astrocytes and microglia, but OPCs within the nerve also have a muted response with reduced proliferative and morphological changes. The lack of strong cellular responses to the loss of oligodendrocytes may be a major factor that contributes to the lack of remyelination in this model.

In previous studies using MBP- iCP9 animals, local delivery of CID to the dorsal spinal cord during the first postnatal week resulted in rapid demyelination in the treated region. Within 2 weeks after CID treatment, the level of myelination had recovered and compared to untreated areas, there was a higher density of CC1+ oligodendrocytes. Furthermore, when subjected to an LPC lesion as an adult, the areas of spinal cord that had been subjected to early oligodendrocyte loss failed to repair as effectively as untreated controls suggesting a lasting effect of the early insult [13]. The rapid remyelination in the early dorsal spinal cord studies following oligodendrocyte apoptosis contrasts with the failure of repair seen in the current optic nerve studies. There are several possible explanations for the different outcomes. While the spinal cord studies were done in early postnatal animals, the optic nerve studies were conducted on older animals and the capacity for myelin repair may diminish with age. It is well known that the capacity for myelin repair is age dependent as analysis of repair of LPC lesions has shown limited repair in aged animals [26]. This seems an unlikely explanation for the relative lack of repair in the optic nerve studies since the animals were less than a month old at the time of the CID delivery, an age at which repair is robust in other models [27, 28]. An alternative possibility is that the method of demyelination affects the capacity for repair. Although there are known differences in myelin repair between lysolecethins [7] and ethidium bromide [9] lesions suggesting that the method of lesion may influence repair, this also seems an unlikely explanation for the differences between optic nerve and spinal cord. In both cases, local delivery of CID to the spinal cord and these studies, oligodendrocyte loss was induced by activation of the iCP9 construct in MBP+ cells. The difference between the models is primarily the mode of CID delivery. In the spinal cord, CID was delivered directly onto the dorsal region while in the optic nerve the CID was delivered indirectly through an intravitreal injection. It may be that direct delivery was a more potent demyelination stimuli and thus provoked a more pronounced repair response. Consistent with this hypothesis, the level of demyelination was greater in the spinal cord than optic nerve. Alternatively, it may be that direct delivery of CID stimulated a subtle traumatic response that promoted enhanced repair in the developing spinal cord.

The relative lack of myelin repair in the optic nerve studies most likely reflects a combination of both the mechanism of induction of demyelination and the location of the lesion. In a model of induction of oligodendrocyte death driven by diptheria toxin, demyelination was delayed for several weeks after oligodendrocyte death and remyelination was extremely slow [29, 30]. Similar to the current study, cell death in this model was induced indirectly, was not associated with rapid microglial or astrocyte responses, and had no direct associated CNS trauma suggesting that these responses are important factors in initiating myelin repair. In addition, several studies using a variety of approaches have suggested that remyelination in the optic nerve may be less efficient than in other regions of the CNS such as the spinal cord and corpus callosum [31]. Why repair in the optic nerve is less effective is unknown. One possibility is that regions that show robust myelin repair are frequently adjacent to a source of neural stem cells such as the subventricular zone or central canal and that these cells contribute to repair. The optic nerve lacks such germinal zone and the adult OPCs in the nerve may be insufficient to mount effective remyelination. Development of robust optic nerve demyelinating/remyelinating models will help address these questions in future studies.

An unexpected finding from the present studies is the patchy nature of the demyelination induced by CID delivery. Labeling of the nerve with antibodies to DsRed did not reveal a non-uniform distribution along the length of the nerve. Double labeling studies with antibodies to DsRed and CC1 suggested that 65–70% of oligodendrocytes expressed DsRed. Based on the pattern of DsRed expression, it was anticipated that there would be uniform loss of oligodendrocytes and myelin along the length of the nerve. One possible reason for the patchy distribution is that the penetration of the CID was not uniform along the nerve and only reached a high enough threshold in some regions to promote cell death. Why there would be a differential distribution of CID penetration in some regions of the nerve is unclear but may be related to the arrangement of vasculature or astroglial organization. A second possibility is that there are different spatially distinct populations of MBP+ cells in the optic nerve that have differential sensitivity to caspase induced cell death. This seems somewhat unlikely although early studies in heterozygous *shiverer* mutant animals demonstrated a chimerism of myelination suggesting there are domains of myelinating oligodendrocytes that are derived from the different genotypes [32]. How the cells are segregated into domains is unclear but may reflect the origin and migration as well as the proliferation of distinct cell populations. Finally, it may be that once an individual oligodendrocyte undergoes apoptosis, it triggers cell death in adjacent oligodendrocytes cells leading to region of demyelination. The notion of cascades of cell death is well established in cell culture and loss of connectivity between adjacent glial cells has been shown to lead to cell death.

A striking feature of the current model is the lack of activation of adjacent astrocytes, microglia and OPCs to the induction of oligodendrocyte apoptosis. In other systems, cells undergoing apoptosis release “eat me” signals that activate adjacent phagocytic cells [33]. In the optic nerve model, we saw little activation of microglia at 2 days post CID when large number of oligodendrocytes are dying. It may be that the signal was not sufficiently strong to stimulate adjacent microglia, although this seems unlikely since the apoptotic debris was rapidly cleared from the nerve. Indeed, it is possible the response of both astrocytes and microglia was sufficiently transient and was missed in the current analysis, or that the canonical responses are related to differential activation of the M1/M2 pathway and not driven by non-traumatic cell apoptosis. In many other models of demyelination driven either by immune or toxin mediated oligodendrocyte loss, there is a rapid and robust proliferative response in adjacent OPCs. By contrast, in the current optic nerve demyelination model, there is only a muted proliferative response in OPCs following oligodendrocyte apoptosis. It is tempting to speculate that the lack of a local microglial or astrocytic response is related to the lack of OPC responses that in turn result in remyelination failure in this model.

In conclusion, we have developed a model of oligodendrocyte death in the murine optic nerve that results in delayed demyelination, a lack of remyelination, and a muted response in adjacent glial cells. Such a model is potentially useful in understanding the cellular and molecular mechanisms that initiate myelin repair and as a platform for assaying the effectiveness of remyelinating therapies.
